# Comparative Genomics Reveals Three Genetic Groups of the Whitefly Obligate Endosymbiont *Candidatus* Portiera aleyrodidarum

**DOI:** 10.3390/insects14110888

**Published:** 2023-11-17

**Authors:** Teng Lei, Ning Luo, Chao Song, Junwei Yu, Yuhang Zhou, Xin Qi, Yinquan Liu

**Affiliations:** 1 Zhejiang Provincial Key Laboratory of Plant Evolutionary Ecology and Conservation, School of Life Sciences, Taizhou University, Taizhou 318000, China; leiteng@tzc.edu.cn (T.L.);; 2 Natural Resources and Planning Bureau of Linhai City, Linhai 317000, China; 3 Ministry of Agriculture and Rural Affairs Key Laboratory of Agricultural Entomology, Key Laboratory of Biology of Crop Pathogens and Insects of Zhejiang Province, Institute of Insect Sciences, Zhejiang University, Hangzhou 310058, China

**Keywords:** comparative genomics, divergence, whitefly obligate endosymbiont, *Portiera*

## Abstract

**Simple Summary:**

Many obligate endosymbionts colonize an invertebrate host and are directly transferred maternally to the host’s embryo. Consequently, the symbiotic bacteria from diverse host species evolve independently, reflecting the host’s phylogeny. Whiteflies, which consist of thousands of species, harbor obligate endosymbionts of the *Portiera* genus. The divergence status of these bacteria, after a long history of coevolution with their hosts, remains ambiguous. In the present study, we aim to unravel the divergence of obligate endosymbionts from different whitefly species through genome comparison. Our findings indicate that these endosymbionts have diverged into at least three disparate genetic groups. Such findings underscore the divergence of whitefly obligate endosymbionts and provide a cue for investigation into the co-divergence between obligate endosymbionts and their hosts.

**Abstract:**

Maternally inherited obligate endosymbionts codiverge with their invertebrate hosts and reflect their host’s evolutionary history. Whiteflies (Hemiptera: Aleyrodidae) harbor one obligate endosymbiont, *Candidatus* Portiera aleyrodidarum (hereafter *Portiera*). *Portiera* was anciently acquired by whitefly and has been coevolving with its host ever since. Uncovering the divergence of endosymbionts provides a fundamental basis for inspecting the coevolutionary processes between the bacteria and their hosts. To illustrate the divergence of *Portiera* lineages across different whitefly species, we sequenced the *Portiera* genome from *Aleyrodes shizuokensis* and conducted a comparative analysis on the basic features and gene evolution with bacterial genomes from five whitefly genera, namely *Aleurodicus*, *Aleyrodes*, *Bemisia*, *Pealius,* and *Trialeurodes*. The results indicated that *Portiera* from *Bemisia* possessed significantly larger genomes, fewer coding sequences (CDSs), and a lower coding density. Their gene arrangement differed notably from those of other genera. The phylogeny of the nine *Portiera* lineages resembled that of their hosts. Moreover, the lineages were classified into three distinct genetic groups based on the genetic distance, one from *Aleurodicus* (Aleurodicinae), one from *Bemisia* (Aleyrodinae), and another from *Aleyrodes*, *Pealius,* and *Trialeurrodes* (Aleyrodinae). Synonymous and nonsynonymous rate analyses, parity rule 2 plot analyses, neutrality plot analyses, and effective number of codons analyses supported the distinction of the three genetic groups. Our results indicated that *Portiera* from distant hosts exhibit distinct genomic contents, implying codivergence between hosts and their endosymbionts. This work will enhance our understanding of coevolution between hosts and their endosymbionts.

## 1. Introduction

Many invertebrates rely on maternally inherited obligate endosymbionts for their viability and fecundity [1,2]. The symbionts colonize hosts’ specialized bacteriocytes and are transferred to hosts’ embryos [3,4]. In such cases, host and symbiont phylogenetic trees are often congruent, indicating cospeciation and synchronous diversification [5,6]. During long-term coevolution with hosts since their transmission from a free-living to an intracellular lifestyle, endosymbiotic bacteria have been exposed to distinct pressure compared with that of free-living ones, leading to an extraordinary genome evolution process [7,8]. Understanding the characteristics of endosymbiont genomes helps reveal the forces that shape the evolution of these bacterial associates.

Due to the intimate association between invertebrates and their obligate endosymbionts, the latter is often used as an auxiliary tool to resolve the phylogenetic relationships underlying the former. The endosymbiont topology of *Candidatus* Evansia muelleri, *Candidatus* Carsonella ruddii, and *Candidatus* Portiera aleyrodidarum was found to be concordant with the topology of their insect hosts Coleorrhyncha, Psylloidea, and Aleyrodidea [9]. Codivergence was also observed between *Diaphorina citri* and its obligate endosymbiont *Candidatus* Carsonella ruddii [10]. The molecular phylogenies inferred from aphid and *Buchnera* genes indicated significant congruence between aphids and *Buchnera* at generic and interspecific levels [11]. Nevertheless, whitefly *Bemisia tabaci* possessed *Portiera* with extensive genome rearrangement [12]. Considering the genomic instability of some *Portiera* lineages, we should be cautious when utilizing this endosymbiont to investigate the phylogeny of whiteflies and the divergence of *Portiera* warrants further studies.

Anciently acquired symbionts exhibit greater genome stability and slower sequence evolution than recently acquired ones. The former exhibit lower synonymous and nonsynonymous substitution rates [13]. Some symbiont lineages from different host species present significant differences with regard to rates of gene evolution [14]. It remains unclear whether genomic differentiation has led to the species differentiation of bacterial lineages from closely related hosts. Investigations of codon usage bias in genomes can reveal phylogenetic relationships between organisms and the molecular evolution of genes [15]. This has been fully investigated in whitefly obligate endosymbionts.

Whitefly (Hemiptera: Aleyrodidae) is a model insect coexisting with its obligate endosymbiont *Portiera*. *Portiera*, which is strictly maternally inherited by whiteflies, has been codiverging with hosts since its origin [16,17]. Complete *Portiera* genomes have been described from *Aleurodicus*, *Bemisia*, *Pealius* and *Trialeurodes* whiteflies [12,14,16,18,19,20]. Comparative genomics revealed that *Bemisia* obligate endosymbionts have lost the ancestral genome order. The genome instability of *Bemisia* obligate endosymbiont might result from the loss of the DNA polymerase III proofreading subunit (*dnaQ*) [16]. The presence and absence of *dnaQ* has been determined in multiple *Portiera* strains. Nevertheless, the category of registered genomes is far from adequate to analyze *Portiera* genome instability and the loss of *dnaQ*. Moreover, codon bias signatures have not been revealed for these genome-stable and genome-unstable lineages.

In this work, we obtained the complete genome of a novel *Portiera* lineage from *Aleyrodes shizuokensis*. This is the first *Portiera* genome from *Aleyrodes*. We analyzed its gene evolution together with other genome-stable and genome-unstable lineages, aiming to reveal the divergence of *Portiera*.

## 2. Materials and Methods

### 2.1. Insect Samples and Genome Sequencing

Whitefly adults were collected from *Oxalis corniculata* in July 2020 in Hangzhou, China (30°18′32″ N, 120°5′49″ E). The specimens were identified as *Aleyrodes shizuokensis* Kuwana from their morphological traits [21] and further confirmed via by *COI* barcode data blasted in NCBI [22]. The insects were kept in 75% ethanol by volume at −80 °C until DNA extraction. Total genomic DNA was extracted from a single female adult using the DNeasy Blood & Tissue Kit (QIAGEN, Valencia, CA, USA) following the manufacturer’s instructions with slight modifications. In brief, the insects were homogenized in 54 µL of Buffer ATL followed by incubation with 6 µL of proteinase K at 56 °C for 6 h to lyse the tissue. Then, equivalent volumes of Buffer AL and ethanol were successively added to the sample. The mixture was centrifuged at 6000× *g* for 1 min at room temperature and washed using 200 µL of Buffer W1 and 200 µL of Buffer W2 successively. Finally, the DNA was eluted with 60 µL of Buffer AE and then stored at −20 °C for later use. The isolated DNA was used for a PCR reaction and whole-genome shotgun sequencing. The *COI* barcode was amplified using universal PCR primers of C1-J-2195 and TL2-N-3014 [23]. PCR was performed in a 20 µL reaction volume, including 1 U rTaq (Takara), 2 µL of 10 × PCR Buffer (Mg^2+^ Plus), 3.2 µL of dNTPs (2.5 mmol/L each), 0.2 µL of each primer (20 µmol/L), 12.2 µL of ddH_2_O, and 2 µL of DNA template. The PCR reaction program was initialized at 94 °C for 3 min, followed by 34 cycles of 96 °C for 30 s, 52 °C for 30 s, 72 °C for 90 s, with a final extension for 10 min at 72 °C. The remaining DNA was fragmented via sonication and used to construct short-insert libraries (insert size of 150 bp) using the Illumina TruSeq Nano DNA Library Preparation Kit. Sequencing was performed on the Illumina HiSeq 4000 platform with a PE150 strategy.

### 2.2. Genome Assembly and Annotation

Raw data generated from the Illumina libraries were trimmed, and the adapters were filtered using Trimmomatic (version 0.39) [24]. The quality of the clean data was assessed using FastQC (version 0.11.9) (https://www.bioinformatics.babraham.ac.uk/projects/fastqc/; last accessed 30 October 2023). To screen out candidate reads belonging to *Portiera*, clean data were mapped to *Portiera* complete genomes available from NCBI (Table 1) using Bowtie2 (version 2.3.5.1) [25]. Draft genomes and those from the same host species were ignored to reduce computational complexity. The selected reads were assembled de novo using SPAdes (version 3.14.1) [26], and the assembled contigs and scaffolds were evaluated using QUAST (version 5.0.2) [27]. To discard sequences not belonging to *Portiera*, a minimum coverage of 200 was required. One scaffold consisting of five contigs was generated. PCR reaction was used to close gaps between contigs. PCR primers and procedures are provided in Table S1. The assembled sequence of the bacterial genome was annotated using Prodigal, RNAmmer, Aragorn, SignalP and Infernal integrated in the Prokka tool [28]. The gene *dnaK* was identified and used to set the coordinates of the genome. Gene synteny between *Portiera* genomes, based on nucleotide sequences, was pairwise analyzed and plotted with the Python package MCscan (https://github.com/tanghaibao/mcscan; last accessed 30 October 2023) [29].

### 2.3. Phylogenetic Relationships

Protein sequence from the *Portiera* genomes were used as input for OrthoFinder (version 2.5.4) [30] to identify orthologous genes. Upset plots of single-copy orthologous genes were drawn by the UpSetR (version 1.4.0) [31] and plotrix (version 3.8-2; https://www.rdocumentation.org/packages/plotrix/versions/3.8-2; last accessed 30 October 2023) packages in R. All single-copy orthologous genes were used for the reconstruction of phylogenetic trees. Sequences of individual genes from the *Portiera* lineages were aligned using MAFFT (version 7.490) [32], followed by eliminating poorly aligned positions using trimAL (version 1.4) [33]. Then, the aligned sequences were concatenated using phylotools package in R (version 0.2.2; https://github.com/helixcn/phylotools; last accessed 30 October 2023). The concatenated datasets were subjected to IQ-TREE (version 2.2.0) for model selection under AICc criterion [34,35]. A maximum likelihood (ML) phylogenetic tree was reconstructed using the inferred model with 1 000 replicates of ultrafast bootstrap in IQ-TREE [36,37]. A Bayesian inference (BI) tree was reconstructed using the inferred model in MrBayes (version 3.2) with a chain length of 10,000,000 [38]. By running BLAST against nr database in NCBI, we chose *Zymobacter palmae* (accession number AP018933) as an outgroup because 16S rRNA genes of *Z. palmae* and *Portiera* had the highest similarity.

To reconstruct the phylogenetic tree of *Portiera* hosts, whitefly mitochondrial genomes were downloaded from NCBI (Table 2). The *COI* gene was used to set the coordinates of the mitochondrial genomes. Then, the nucleotide sequences were aligned using MAFFT and trimmed using trimAL. Model selection and phylogenetic tree reconstruction methods resembled those of *Portiera*. *Diaphorina citri* (accession number NC_030214) [39] was used as an outgroup because of its close relationship with whiteflies [9].

### 2.4. Genetic Distance

The genetic distance of *Portiera* lineages was evaluated using three indicators, the average nucleotide identity (ANI), the amino acid identity (AAI) and Mash distance. The ANI and AAI values of the single-copy orthologous genes were pairwise-compared using the fastANI package in R [46] and the EzAAI package in Java [47]. Visualization was performed using the R package corrplot. Mash distance values and a Mash-based phylogenetic tree were estimated based on concatenated datasets using Mashtree (version 1.2.0) with 1 000 replicates of ultrafast bootstrap [48]. Based on the results of genetic distance analysis, the nine *Portiera* lineages were divided into three genetic groups. Group BM consisted of *Portiera* from *B. tabaci* MEAM1 (BTB), *B. tabaci* MED (BTQ), *B. tabaci* Asia II 3 (BTZ1) and *B. tabaci* China1 (BTZ3). Group AD consisted of *Portiera* from *Aleyrodes shizuokensis* (AdSh), *P. mori* (PeMo) and *T. vaporariorum* (TrVa). Group AL consisted of *Portiera* from *Aleurodicus dispersus* (AlDi) and *Aleurodicus floccissimus* (AlFl). The whiteflies’ Mash distance based on mitochondrial genomic data was also estimated using Mashtree with default parameters.

### 2.5. Codon Usage Bias

The codon usage bias of three *Portiera* genetic groups was comparatively analyzed. The *Portiera* of the *B. tabaci* cryptic species complex presented the highest ANI and AAI values. One of them, BTZ3, was used as a reference to evaluate nucleotide substitution rates. *Ka* indicated the rate of nonsynonymous nucleotide substitution per nonsynonymous site. *Ks* indicated the rate of synonymous substitution per synonymous site. *Ka* and *Ks* values were calculated using the KaKs_Calculator 2.0 toolkit [49]. Bias in the third codon letters of each genome or genetic group was analyzed using a parity rule 2 (PR2) bias plot. A3, T3, C3, and G3 indicated the nucleotide content of A, T, C, and G, respectively, at the third base of codons in each gene. Their values were calculated using CodonW (version 1.4.4) (https://sourceforge.net/projects/codonw/; last accessed 30 October 2023). In PR2 bias plot maps, G3/(G3 + C3) and A3/(A3 + T3) were set as the abscissa and ordinate, respectively [50]. The GC content of the first, second and third bases of codons (GC1, GC2 and GC3) for each gene was calculated in Python, and a linear regression of GC12 and GC3 was performed in R to conduct a neutrality plot analysis, where GC12 represented the average of GC1 and GC2 [51]. To reveal the relationship between nucleotide composition and codon bias, the GC content of the third synonymous position (GC3s) and effective number of codons (ENC) generated by CodonW were used to conduct ENC plot analysis. In ENC plot maps, ENC values were compared to the theoretical curve ENC_exp_ = 2 + GC3s + 29/[GC3s^2^ + (1 − GC3s)^2^] [52].

## 3. Results

### 3.1. The Genomic Features of Portiera

**Figure 1:**
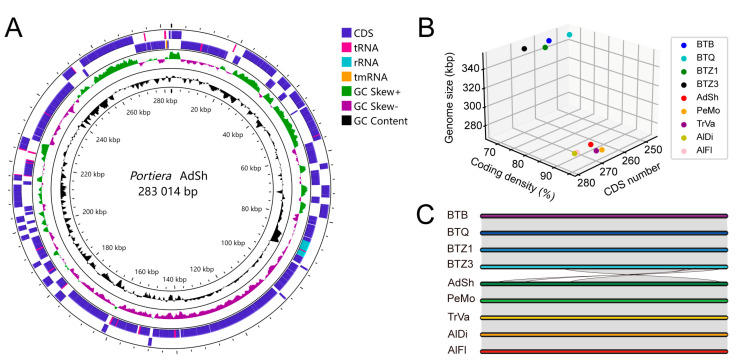
Genome features of *Portiera* lineages. (**A**) Circular map of *Portiera* AdSh genome. From outer to inner: genes, GS skew, GC content, and scale bar. (**B**) Diagram of *Portiera* genome size, coding density and coding sequences (CDSs). BTB, BTQ, BTZ1, BTZ3, AdSh, AlDi, AlFl, PeMo and TrVa represent the *Portiera* lineages from *Bemisia tabaci* MEAM1, *B. tabaci* MED, *B. tabaci* Asia II 3, *B. tabaci* China1, *Aleyrodes shizuokensis*, *Aleurodicus disperses*, *Aleurodicus floccissimus*, *Pealius mori* and *Trialeurodes vaporariorum*, respectively. (**C**) *Genome synteny* of *Portiera* lineages. Genomic protein-coding nucleotide sequences were used to conduct synteny analyses. The synteny was pairwise-analyzed, and the results were merged in one figure.

The genome of AdSh from *A. shizuokensis* is composed of a single circular chromosome with an average coverage of 317×. The genome is 283,014 bp in length and contains 268 coding sequences (CDSs), three rRNAs, 34 tRNAs, one tmRNA and one RnaseP RNA. It exhibits a low GC content (23.8%) and a high coding density (91.4%) (Figure 1A). Functional *dnaQ* is observed in AdSh genome. These features are roughly similar to those of previously sequenced *Portiera* genomes except those of the *Bemisia tabaci* cryptic species complex [16]. Based on the basic genomic features of *Portiera*, the nine genomes are clustered into two groups. Genomes of BTB, BTQ, BTZ1 and BTZ3 from *B. tabaci* present lower coding density (67.6–67.9%), fewer CDSs (247–267) and larger sizes (349.1 kb–350.1 kb). Genomes from the other whiteflies present higher coding density (91.7–94.9%), more CDSs (266–279) and smaller sizes (271.2 kb–283.6 kb) (Figure 1B). Synteny analysis reveals two *Portiera* gene arrangement patterns, one from *B. tabaci* and the other from other whiteflies (Figure 1C). *Portiera* lineages from *B. tabaci* have lost the ancestral genome order as described by Santos-Garcia et al., 2020 [16].

### 3.2. The Phylogeny of Portiera and Hosts

**Figure 2:**
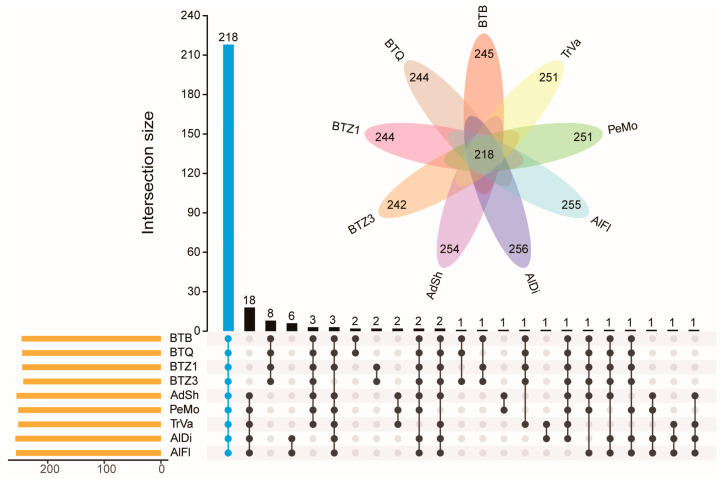
UpSet plot of orthogroups from *Portiera* genomes. The orthogroups present in all lineages are colored blue. Those present in partial lineages are colored black. The numbers of orthogroups in each lineage are indicated in the Venn diagram.

A total of 218 orthogroups were present in all *Portiera* lineages, among which 198 were single-copy orthogroups. Eighteen orthogroups were absent in the *Portiera* of *B. tabaci*, and eight were present only in those of *B. tabaci*. Six orthogroups were present only in those of Aleurodicinae and three only in those of Aleyrodinae (Figure 2). The results indicated that the *Portiera* of *B. tabaci* differed from those of other whiteflies in gene content. And the *Portiera* of Aleurodicinae differed from those of Aleyrodinae.

**Figure 3:**
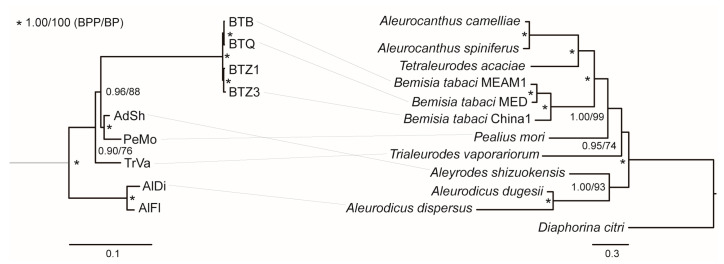
Bayesian inference (BI) and maximum likelihood (ML) trees inferred from *Portiera* single-copy orthologous genes (**left**) and whiteflies’ mitochondrial genomes (**right**). Topologically consistent BI and ML trees are integrated. Bayesian posterior probabilities (BPP) and ultrafast bootstrap support values (BP) are given at the nodes. *Portiera* lineages and corresponding host species are connected by straight lines. *Zymobacter palmae* AP018933 is used as the outgroup for *Portiera* but is not displayed for plotting reasons.

BI and ML trees were obtained using *Portiera* single-copy orthologous genes. The two trees shared a common topology where *Portiera* formed four major clades. One clade consisted of AlDi and AlFl, the endosymbionts of Aleurodicinae. Other clades consisted of endosymbionts of Aleyrodinae. This topology was consistent with the tree based on two rRNA (16S and 23S) and three coding genes (*groEL*, *rpoD*, and *dnaK*) [16]. BI and ML trees were also inferred based on whiteflies’ mitochondrial genomes. Similarly, *Aleurodicus dispersus* and *Aleurodicus dugesii* from Aleurodicinae formed one clade, and most Aleyrodinae species formed another huge clade. Intriguingly, *Aleyrodes shizuokensis* from Aleyrodinae was found to be closely related to Aleurodicinae clade. Despite this species and its *Portiera* endosymbiont, *Portiera*-based and mitogenome-based trees were mostly consistent in topology (Figure 3). The divergence between the two topologies implied that the evolution of endosymbionts and hosts might not be completely parallel.

### 3.3. Genetic Distance

**Figure 4:**
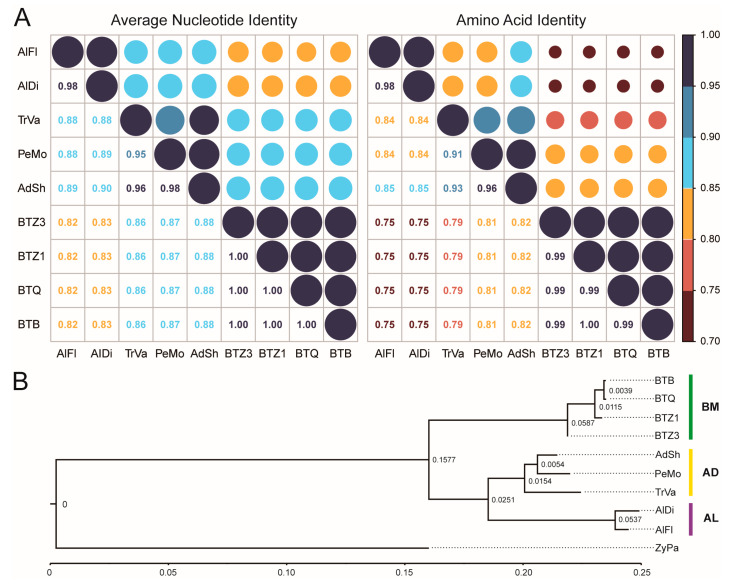
Genetic distance of *Portiera* lineages. (**A**) Matrix diagram of average nucleotide identity (ANI) and amino acid identity (AAI) among *Portiera* genomes. The values of ANI and AAI are shown by both scores and circles, where the values are in accordance with the area of circles and the element colors according to the legend. (**B**) Mash-based phylogenetic tree derived from nine *Portiera* lineages. ZyPa, *Zymobacter palmae* AP018933, is used as outgroup to root the tree. Branch lengths representing Mash distance values are indicated at the nodes.

The results of ANI and AAI analysis indicated that bacterial genome similarities were mostly congruous with their insect hosts, except for *B. tabaci* endosymbionts. Specifically, phylogenetically closely related hosts harbored endosymbionts with high ANI values (AlDi vs. AlFl, 0.98; AdSh vs. PeMo, 0.98; AdSh vs. TrVa, 0.96; PeMo vs. TrVa, 0.95) and AAI values (AlDi vs. AlFl, 0.98; AdSh vs. PeMo, 0.96; AdSh vs. TrVa, 0.93; PeMo vs. TrVa, 0.91). Between symbionts from distantly related Aleurodicinae and Aleyrodinae hosts, the ANI and AAI values were no more than 0.90 and 0.85, respectively. Additionally, *B. tabaci* cryptic species harbored endosymbionts with the highest ANI and AAI values, both over 0.99. Although *B. tabaci* belonged to Aleyrodinae, their endosymbionts shared low ANI and AAI values with other Aleyrodinae endosymbionts (ANI, 0.86–0.88; AAI, 0.79–0.82). The values were even lower than those shared by other Aleyrodinae and Aleurodicinae endosymbionts (ANI, 0.88–0.90; AAI, 0.84–0.85) (Figure 4A). The ANI and AAI of shared genes between two prokaryotes provide a robust means to assess genetic relatedness among lineages. ANI and AAI values over 95% are necessary conditions for species definition in prokaryotes [53,54]. In this study, due to the lack of other sufficient conditions for species definition, we used the criteria ANI > 95% and AAI > 90% to divide the *Portiera* lineages into three genetic groups, one from Aleurodicinae whiteflies (hereafter AL) and two from Aleyrodinae whiteflies. Aleyrodinae whiteflies harbor two distinct *Portiera* genetic groups, one from the *Bemisia* genus (hereafter BM) and the other from other genera (hereafter AD). Mash distance analysis supported the division of the three genetic groups. The within-group distance (0.0017–0.0454) was much shorter than the between-group distance (0.0889–0.1766) (Figure 4B). Additionally, the *Portiera* genomic distance was much shorter than whiteflies’ mitogenomic distance, indicating a slower divergence rate of *Portiera* lineages than their hosts (Table S2).

### 3.4. Codon Usage Bias

#### 3.4.1. Synonymous and Nonsynonymous Substitution Rates

**Figure 5:**
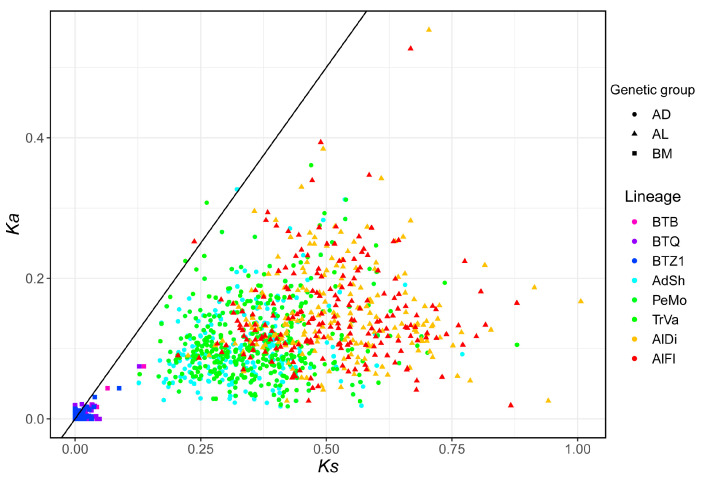
Distribution of *Ka* and *Ks* values in *Portiera* genomes. *Ka*, the rate of nonsynonymous nucleotide substitution per nonsynonymous site; *Ks*, the rate of synonymous substitution per synonymous site. *Portiera Ka* and *Ks* values are calculated with reference to BTZ3. *Portiera* lineages are distinguished by plot colors, and genetic groups are distinguished by plot shapes.

The *Ka* and *Ks* values were used to test for signatures of natural selection. This index was used to assess the difference in selective pressure by *Portiera* genetic group and lineage. Taking BTZ3 as a reference, most genes in the BM group have not undergone synonymous or nonsynonymous mutations. The *B. tabaci* cryptic species diverged recently, the other whitefly species diverged a long time ago [16]. Mutations might have no time to occur or become fixed in *Portiera* from *B. tabaci* species while that happened in the other species. In AD and AL groups, *Ka* values were observed to be much smaller than *Ks* values for most of the genes, indicating the substantial influence of purifying selection (Figure 5). Compared with AD group, AL exhibited elevated synonymous and nonsynonymous substitution rates (Table S3). The results supported the divergence of the three genetic groups we defined.

#### 3.4.2. PR2 Bias Analysis

**Figure 6:**
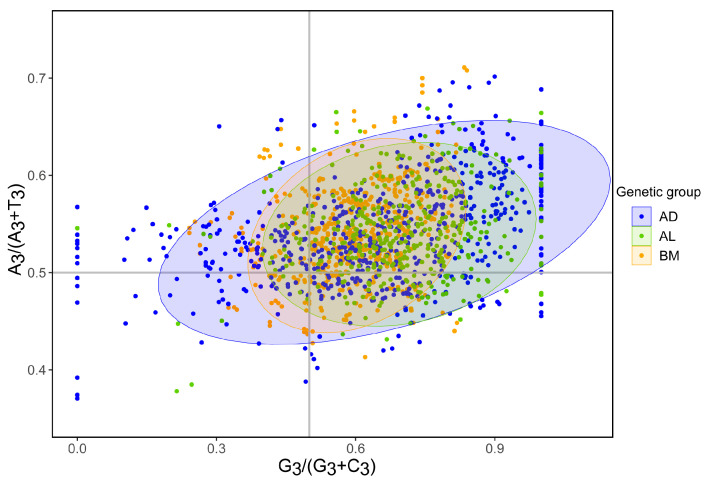
Parity rule 2 (PR2) bias plot analysis of *Portiera* genetic groups. A3, T3, C3, and G3 indicate the nucleotide contents of A, T, C, and G, respectively, at the third base of codons in each gene. The genes are plotted on the graphs with G3/(G3 + C3) on the abscissa and A3/(A3 + T3) on the ordinate. The graphs and plots are divided into four quadrants by the lines G3/(G3 + C3) = 0.5 and A3/(A3 + T3) = 0.5. The oval shadows represent the 95% confidence interval. PR2 bias plot analysis of individual lineages is provided in Figure S1.

Bias in the third codon letters of each genetic group or genome was analyzed using PR2 bias analysis to evaluate their genetic difference. In the PR2 bias plot graphs, G3/(G3 + C3) and A3/(A3 + T3) were set as the abscissa and ordinate, respectively. The graphs are divided into four quadrants by the lines G3/(G3 + C3) = 0.5 and A3/(A3 + T3) = 0.5. The majority of gene points are distributed in the quadrants G3/(G3 + C3) > 0.5 and A3/(A3 + T3) > 0.5, indicating significant G and A bias at the third codon letter (Figure 6 and Figure S1). Furthermore, the distribution patterns of genes varied among *Portiera* genetic groups. The plots of the BM group were the most concentrated, while those of the AL group were the most scattered in the graph, implying that PCGs of the BM group were stricter with the content of bases at the third position of the codons.

#### 3.4.3. Neutrality Plot Analysis

**Figure 7:**
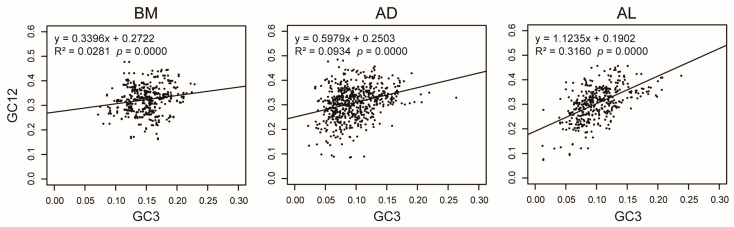
Neutrality plot analysis of *Portiera* genetic groups. GC3, GC content of the third base of the codons in each gene; GC12, average GC content of the first and second bases of the codons in each gene. The values of GC3 and GC12 are indicated at the abscissae and ordinates, respectively. Linear regression analysis between GC12 and GC3 was performed using R. Neutrality plot analysis of individual lineages is provided in Figure S2.

Neutrality plot analysis was performed to estimate the extent of gene directional mutation pressure against selection. The results helped to reveal the genetic diversification of *Portiera* genetic groups and lineages. Linear regression analysis between GC12 and GC3 indicated that correlation coefficients (R^2^) were low in all the genetic groups. This implies that there is no significant correlation between GC12 and GC3. Thus, the influence of natural selection on codon usage bias outweighs the impact of mutation. The correlation coefficient of the BM group (R^2^ = 0.02181) was the lowest among the three genetic groups, implying that GC12 and GC3 of BM genes were the least correlated (Figure 7 and Figure S2). Thus, BM group suffered more selection than the AD and AL groups.

#### 3.4.4. ENC Plot Analysis

**Figure 8:**
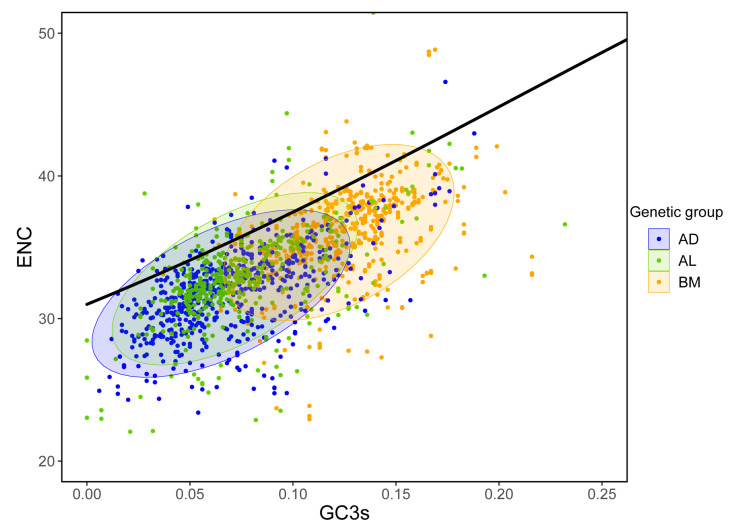
Effective number of codons (ENC) plot analysis of *Portiera* genetic groups. GC3s indicates the GC content at the third synonymous position. ENC_exp_ indicates the theoretical ENC value. The continuous curve ENC_exp_ = 2 + GC3s + 29/[GC3s^2^ + (1 − GC3s)^2^] represents the relationship between ENC_exp_ and GC3s under H_0_ (no selection). The oval shadows represent the 95% confidence interval. ENC plot analysis of individual lineages is provided in Figure S3.

ENC plot analysis was used to investigate the codon usage patterns across the *Portiera* genetic groups and lineages to reveal their genetic diversification. The relationship between the theoretical ENC value (ENC_exp_) and GC3s under H_0_ (no selection) was approximated by ENC_exp_ = 2 + GC3s + 29/[GC3s^2^ + (1 − GC3s)^2^]. Points beneath the curve by more than a 5% error margin imply selection pressure on the genes. In the graphs, most of the points are below the curve for all the genetic groups, indicating selection pressure on these genes (Figure 8 and Figure S3). The BM group was found to possess the highest GC3s and ENC values. Nevertheless, there was no obvious difference between the AD and AL groups (Table S4). That was probably because the divergence among them was not large enough to be distinguished via the ENC plot analysis. *Buchnera* lineages displaying notable variation in the PR2 analysis also exhibited comparable ENC analysis results, indicating a lack of discernible differences [8].

## 4. Discussion

The whitefly obligate endosymbiont *Portiera* established symbiosis with its host before the origin of Aleyrodidae and has been codiverging with whiteflies ever since [16]. Because of the strict mother-to-offspring transmission, *Portiera* does not switch to other host species [55]. Thus, *Portiera* reflect both their own and their host’s phylogenetic relationships [44]. In this work, the phylogenetic tree inferred from bacterial single-copy orthologous genes was consistent with their hosts’ phylogenetic relationships. In contrast, many facultative endosymbionts have been demonstrated to be horizontally transmitted, leading to the same symbiont residing in multiple host species [56,57]. Furthermore, although it has not been reported, one facultative endosymbiont might be horizontally transferred to another host and then back to its former host. Transferable facultative endosymbionts coexist with multiple hosts and experience a shorter codivergence period with their present host; thus, their phylogeny cannot reflect that of their hosts [57]. Relative to facultative endosymbionts, maternally inherited obligate endosymbionts are more reliable for the phylogenetic analysis of insect hosts.

The genome evolution of endosymbionts is shaped by different selection intensities that may reflect the different ages and metabolic roles of symbiont types. Compared with the recently acquired sharpshooter symbiont *Candidatus* Baumannia cicadellinicola, the anciently acquired *Candidatus* Sulcia muelleri exhibits much greater genome stability and slower sequence evolution [13]. Intraspecific genetic variation in hosts also affects the regulation of obligate symbionts, leading to lineage-specific patterns of genome evolution [58,59]. *Portiera* is an anciently acquired endosymbiont of the whitefly and has codiverged with its host for millions of years. Our results indicated that *Portiera* genomes are stable in size, gene content and gene order, except those from *B. tabaci*. *Portiera* of *B. tabaci* formed a distinct genetic group. The genome instability of the *B. tabaci* endosymbiont might result from the absence of *dnaQ* [16]. We assembled a *Portiera* genome from the *Aleyrodes* genus, which contains *dnaQ*, and found that the general genome features were similar to those of other genome-stable *Portiera* lineages. Previous suggestions that *dnaQ* might be associated with *Portiera* genome instability were sustained.

Maternally inherited obligate endosymbionts codiverge with insect hosts. During the process of host species differentiation, it is unknown whether the symbiotic bacteria split into different species accordingly. Differences in genome size among symbiont lineages from closely related host species have been reported for *Buchnera* of aphids, *Blochmannia* of carpenter ants, and *Portiera* of whiteflies [60,61]. In this work, we compared general genomic features, genetic distance, and codon usage bias to assess the genetic variation in *Portiera*. Based on these results, three *Portiera* genetic groups, AL, AD, and BM, were defined. However, the variation was not qualified to define a new species.

Codon usage bias patterns of *Buchnera* genomes from different aphid subfamily varied. Pemphigidae possessed *Buchnera* with a more complete loss of codon bias, stronger strand asymmetry, and more reduced genome than Aphididae does [62]. The *Buchnera* codon usage pattern mostly resulted from mutation pressure [8]. In this study, mutation was also found to play a crucial role in shaping the codon usage of the three *Portiera* genetic groups we defined, while the BM group suffered more selection than the AD and AL groups. Obvious codon usage bias differences were observed among the genetic groups. Synonymous and nonsynonymous nucleotide substitution rates within genetic groups were lower than those between the genetic groups. The data in this study are necessary but not sufficient to define the genetic groups as species. However, we can tell that *Portiera* species differentiation is slower than that of their hosts.

## 5. Conclusions

We provided a complete genome of *Portiera*, the obligate endosymbiont of *Aleyrodes shizuokensis*. Comparative genomics was performed with eight other *Portiera* genomes. Our results revealed that these *Portiera* lineages have differentiated into at least three genetic groups, one of which resulted from the loss of stability during its evolutionary history. Obligate endosymbionts reflect their hosts’ evolutionary history. The divergence of endosymbionts should be investigated to further illustrate the speciation of their hosts.

## Figures and Tables

**Table 1:** Complete *Candidatus* Portiera aleyrodidarum genomes used in this study.

Portiera ID	Host Subfamily	Host Species	Accession Number ^1^	Reference
BTB	Aleyrodinae	*Bemisia tabaci* MEAM1	CP003708	[19]
BTQ	Aleyrodinae	*Bemisia tabaci* MED	CP003835	[18]
BTZ1	Aleyrodinae	*Bemisia tabaci* Asia II 3	CP016327	[20]
BTZ3	Aleyrodinae	*Bemisia tabaci* China1	CP016343	[20]
AdSh	Aleyrodinae	*Aleyrodes shizuokensis*	GWHBOVO00000000 ^2^	This study
PeMo	Aleyrodinae	*Pealius mori*	LR744089	[16]
TrVa	Aleyrodinae	*Trialeurodes vaporariorum*	CP004358	[12]
AlDi	Aleurodicinae	*Aleurodicus dispersus*	LN649255	[14]
AlFl	Aleurodicinae	*Aleurodicus floccissimus*	LN734649	[14]

^1^ The genomes are deposited at GenBank of NCBI unless otherwise stated. ^2^ The genome of AdSh is deposited at the National Genomics Data Center, China National Center for Bioinformation, Chinese Academy of Sciences.

**Table 2:** Whitefly mitochondrial genomes used in this study.

Subfamily	Species	Accession Number	Reference
Aleyrodinae	*Bemisia tabaci* MEAM1	KR559508	NA ^1^
Aleyrodinae	*Bemisia tabaci* MED	JQ906700	[40]
Aleyrodinae	*Bemisia tabaci* China1	KR559506	NA ^1^
Aleyrodinae	*Aleurocanthus camelliae*	KU761949	[41]
Aleyrodinae	*Aleurocanthus spiniferus*	KJ437166	[42]
Aleyrodinae	*Aleyrodes shizuokensis*	MT880225	[43]
Aleyrodinae	*Pealius mori*	LR877884	NA ^1^
Aleyrodinae	*Tetraleurodes acaciae*	AY521262	[44]
Aleyrodinae	*Trialeurodes vaporariorum*	AY521265	[44]
Aleurodicinae	*Aleurodicus dispersus*	KR063274	[45]
Aleurodicinae	*Aleurodicus dugesii*	AY521251	[44]

^1^ NA, reference not available; downloaded from NCBI.

## Data Availability

The data supporting the findings of this study are openly available from the National Genomics Data Center, China National Center for Bioinformation (CNCB-NGDC), Chinese Academy of Sciences at https://ngdc.cncb.ac.cn (accessed on 16 November 2023) [63]. The complete genome of *Aleyrodes shizuokensis* obligate endosymbiont *Portiera* lineage AdSh has been deposited in CNCB-NGDC under accession number GWHBOVO00000000. The associated BioProject, BioSample and GSA accession numbers are PRJCA012377, SAMC916243 and CRA008827.

## Supplementary Materials

The following supporting information can be downloaded at: https://www.mdpi.com/article/10.3390/insects14110888/s1, Table S1: PCR primers and procedures for completing AdSh genome; Table S2: Mash distance of *Portiera* genomes and whitefly mitochondrial genomes; Table S3: Synonymous and nonsynonymous substitution rates of *Portiera* genomes; Table S4: Codon nucleotide content and ENC values of *Portiera* genomes; Figure S1: Parity rule 2 bias plot analysis of *Portiera* lineages; Figure S2: Neutrality plot analysis of *Portiera* lineages; Figure S3: Effective number of codons plot analysis of *Portiera* lineages.
