# Supplementary figures and images for: Comparative Genomics Reveals Three Genetic Groups of the Whitefly Obligate Endosymbiont Candidatus Portiera aleyrodidarum
Source: Insects. 2023 Nov 17;14(11):888. doi: 10.3390/insects14110888 (PMC10672337; doi:10.3390/insects14110888)

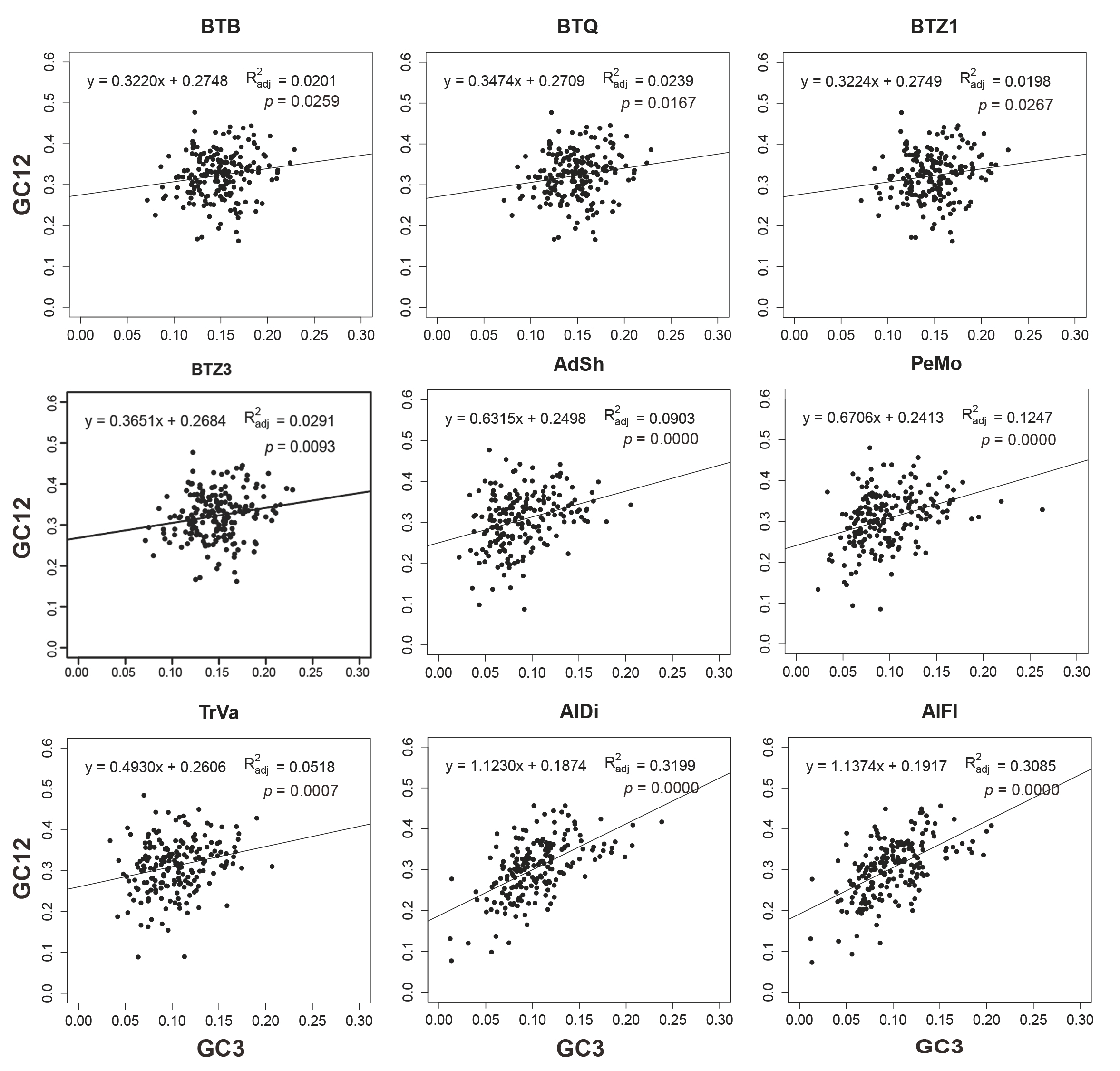

Supplement: Supplementary file 1 [file insects-14-00888-s001.zip › Figure S2.tiff]

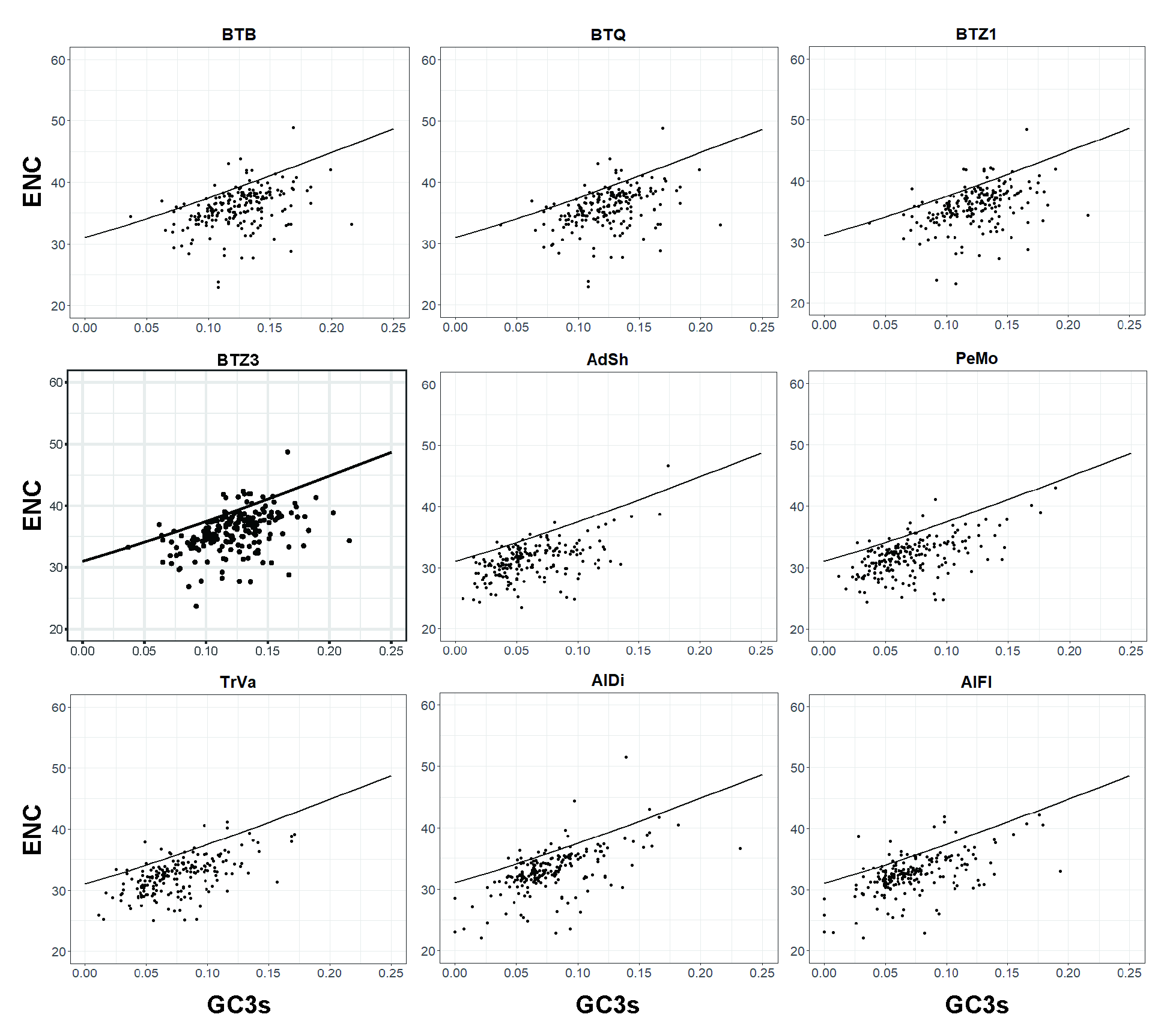

Supplement: Supplementary file 1 [file insects-14-00888-s001.zip › Figure S3.tif]
